# Effect of Different Stages of Chronic Kidney Disease and Renal Replacement Therapies on Oxidant-Antioxidant Balance in Uremic Patients

**DOI:** 10.1155/2013/358985

**Published:** 2013-12-12

**Authors:** Hadja Fatima Tbahriti, Abbou Kaddous, Malika Bouchenak, Khedidja Mekki

**Affiliations:** ^1^Laboratoire de Nutrition Clinique et Métabolique, Faculté des Sciences de la Nature et de la Vie, Université d'Oran, 31100 Oran, Algeria; ^2^Service de Néphrologie, Etablissement Hospitalier Universitaire (EHU) d'Oran, 31037 Oran, Algeria

## Abstract

Oxidative stress seems to be involved in the path physiology of cardiovascular complications of chronic kidney disease (CKD). In this study, we determined the effect of different stages of CKD and substitutive therapies on oxidative stress. One hundred sixty-seven patients (age: 44 ± 06 years; male/female: 76/91) with CKD were divided into 6 groups according to the National Kidney Foundation classification. Prooxidant status was assessed by assaying thiobarbituric acid reactive substances, hydroperoxides, and protein carbonyls. Antioxidant defence was performed by analysis of superoxide dismutase, catalase, glutathione peroxidase, glutathione reductase, vitamin E, Iron, and bilirubin. TBARS and LPO were higher in HD patients compared to other groups (*P* < 0.001), while protein carbonyls were more increased in PD patients. The antioxidant enzymes were declined already at severe stage of CKD and they were declined notably in HD patients (*P* < 0.001). Similar observation was found for vitamin E, Fe, and bilirubin where we observed a significant decrease in the majority of study groups, especially in HD patients (*P* < 0.001). The evolution of CKD was associated with elevated OS. HD accentuates lipid, while PD aggravates protein oxidation. However, the activity of antioxidant enzymes was altered by impaired renal function and by both dialysis treatments.

## 1. Introduction

Cardiovascular diseases (CVD) constitute the major risk of morbidity and mortality in chronic kidney disease (CKD) patients [1, 2]. Uremic patients have both traditional cardiovascular (CV) risk factors (i.e., old age, hypertension, diabetes, smoking, dyslipidemia, heart failure, and physical inactivity) and nontraditional CV risk factors, including malnutrition, anemia, hyperhomocysteinemia, neuropathy, hyperparathyroidism, and chronic inflammation [3–5]. Patients with end-stage renal disease (ESRD) undergoing renal replacement therapy (RRT), either hemodialysis (HD) or peritoneal dialysis (PD), may face a partial loss of some low-molecular-weight plasma factors (i.e., vitamins A, C, and E) [6, 7] that normally contrast inflammation by neutralizing reactive oxygen species (ROS) [8]. Indeed, the latter are increased during the two therapies [9]. The imbalance in antioxidant and pro-oxidant factors generates an oxidative stress (OS) that increases the inflammatory state already present in these patients.

In recent years, OS has been postulated to be an important risk factor for CVD [10]. OS results from an imbalance between prooxidant and antioxidant defence and mechanisms with increased levels of prooxidants leading to tissue damage [10]. Antioxidants can be divided into intracellular and extracellular antioxidants. Intracellular enzymatic antioxidants are superoxide dismutase (SOD), catalase (CAT), and glutathione peroxidase (GSH-GPx), which convert substrates (superoxide anion radicals and hydrogen peroxide) to less reactive forms. Various extracellular antioxidants, such as reduced glutathione (GSH), bilirubin, uric acid, and iron (Fe), prevent free radical (FR) reaction by sequestering transition metal ions by chelation in plasma [10].

Many studies have demonstrated increased OS in patients with CKD, including accumulation of reactive carbonyl compounds as markers of elevated protein peroxidation [10–12] and increased concentrations of thiobarbituric acid reactive substances (TBARS), malondialdehyde (MDA), and Hydroperoxides (LPO) as markers of high lipid peroxidation [13, 14].

OS is particularly detrimental in patients receiving HD after each dialysis session, due to the contact of blood with dialysis membrane, facing a chronic deficit in antioxidant defense system [12].

Thus, this study was undertaken in order to determine the effect of different stages of CKD, hemodialysis, and peritoneal dialysis on lipid peroxidation, protein oxidation, and antioxidant defence.

## 2. Subjects and Methods

### 2.1. Patients

The study was carried out in 167 patients with CKD. Patients were divided into 6 groups according to the classification of the National Kidney Foundation (NKF) [15]. They included 28 patients with GFR equal to 86.28 mL/min (CKD 1), 28 patients with GFR equal to 46 mL/min (CKD 2), 28 patients with GFR equal to 20.31 mL/min (CKD 3), 18 patients with GFR equal to 10.75 mL/min (CKD 4), 40 hemodialysis (HD) patients, and 25 peritoneal dialysis (PD) patients (Table 1).

We excluded from our study patients with clinical signs of infection (hepatitis B and hepatitis C), malignancy, active immunological diseases, and immunosuppressive or immunomodulatory and anti-inflammatory drugs (i.e., conditions that conceal cytokine release). We also excluded patients with diabetes and nephrotic syndrome because diabetes could induce an inflammatory response and rise OS. None of the patients were taking lipid-lowering drugs or antioxidant supplements. Patients take antihypertensive drugs, calcium, vitamin D, and erythropoietin.

The etiology of CKD in our study included hypertension (66%), cystic kidney disease (14%), glomerulonephritis (6%), prostatic obstruction (3%), and pulmonary tuberculosis treatment (1%) and 10% were unknown.

We calculate the creatinine clearance from the serum creatinine and through the following estimation formula: the Cockroft and Gault [16] formula [GFR (1/4) (140 − age) BW × 1.23/creatinin]. In women, this value was multiplied by 0.85. HD patients were on standard bicarbonate using polysulfone membrane. Patients were dialyzed from 14 to 109 months, three times a week, and each session lasting 4 h. PD patients were in dialysis from 05 to 49 months, using a standard procedure (four exchanges: three isotonic 1.36% glucose solutions, then a hypertonic one at 3.86% glucose). We note that the patients' nutrition contains small amounts of protein and phosphate.

All patients were treated at the nephrology ward of the University Hospital of Oran. The purpose of this study was explained to the subjects and the investigation was carried out with their consent. The experimental protocol was approved by the Committee for Research on Human Subjects of Oran.

### 2.2. Assays

In all patients, blood samples were drawn after 12-hour overnight fast from antecubital venipuncture in uremic and PD patients and by the dialysis fistula in HD patients. We used vacutainer tubes with different anticoagulants during the blood samples. Tubes containing the lithium heparin are used for biochemical experiments, and those containing ethylene diamine tetraacetic acid (EDTA) (8%) are used for the hematology analysis. We collected plasma according to the technique low speed centrifugation at 3000 ×g at 4°C for 15 min. The fresh plasma was removed, aliquoted, and stored at −20°C.

#### 2.2.1. Lipid and Protein Peroxidation

Lipid peroxidation was estimated by measuring thiobarbituric acid reactive substances (TBARS) and hydroperoxides (LPO). TBARS concentrations were measured according to the method of Quintanilha et al. [17], using tetramethoxypropane (Prolabo) as precursor of malondialdehyde (MDA). One milliliter of diluted plasma (protein concentration about 2 mg/mL) was added to 2 mL of thiobarbituric acid (final concentration, 0.017 mmol/L) and butylated hydroxytoluene (concentration, 3.36 mmol/L) and incubated for 15 min at 100°C. After cooling and centrifugation, the absorbance of supernatant was measured at 535 nm. Data were expressed as mmol of TBARS produced/mL of sample.

The plasma was also assayed to determine LPO with an assay kit from Cayman Chemical (cat. no. 705003); the rate of increase in the absorbance at 500 nm is directly proportional to the LPO produced.

Oxidized proteins were estimated by measuring carbonyls concentrations. The latter were analyzed in plasma with an assay kit from Cayman Chemical (cat. no. 10005020); the absorbance of the samples was measured between 360 and 385 nm by a plate reader.

#### 2.2.2. Antioxidant Measurements

Activity of antioxidant enzymes was measured in erythrocyte. Superoxide dismutase (SOD; EC 1.15.1.1) activity was determined with Sigma Chemical kits (cat. no. 19160) at 450 nm by measuring the dismutation of superoxide radicals generated by xanthine oxidase and hypoxanthine. One unit was defined as the amount of enzymes necessary to produce 50% inhibition in the rate of p-iodonitrotetrazolium reduction.

Catalase (CAT; EC 1.11.1.6; 2H_2_O_2_ oxidoreductase) activity was measured with an assay kit from Cayman Chemical (cat. no. 707002). CAT is involved in the detoxification of hydrogen peroxide (H_2_O_2_). CAT enzyme activity could be determined by using the peroxidatic function of CAT at 540 nm.

Glutathione peroxidase (GSH-Px; EC 1.11.1.9) enzyme activity was measured with an assay kit from Sigma Chemical (cat. no. CGP1). GSH-Px activity was measured indirectly by a coupled reaction with glutathione reductase (GSH-GR). Oxidized glutathione (GSSG), produced upon reduction of an organic hydroperoxide by GSH-Px, was recycled to its reduced state by GSH-GR and NADPH. The oxidation of NADPH to NADP+ is accompanied by a decrease in absorbance at 340 nm and is indicative of GSH-GPx activity.

Glutathione reductase (GSH-GR; EC 1.6.4.2) enzyme activity was determined with an assay kit from Sigma Chemical (cat. no. GRSA). GSH-GR catalyzed the reduction of GSSG to reduced glutathione (GSH). GSH-GR activity can be measured either by the decrease in absorbance caused by the oxidation of NADRH at 340 nm or by the increase in absorbance caused by the reduction of DTNB at 412 nm.

For the vitamin E (Vit E) we measured it in plasma with an assay kit from Cayman Chemical (cat. no. 10010621). The absorbance of samples was measured between 405 and 420 nm by a plate reader. The plasma concentrations of the bilirubin and iron (Fe) were estimated with a HumaStar 600 automatic analyzer (Human, Germany).

### 2.3. Statistical Analysis

Statistical analysis was performed using SPSS 20.0 (IBM SPSS statistics; USA). Data were expressed as the mean ± SD (standard deviation). The distribution of variables was compared by the *χ* ^2^ analysis. Difference between the arithmetical averages was assessed by ANOVA, which was adjusted for multiple comparisons. Depending on the normality of distribution of variables, the comparisons between groups were performed using one-way of analysis of variance (ANOVA) or the Mann-Whitney *U*-test when results are nonparametrically distributed. All statistical tests were two tailed, and a *P* value below 0.05 was considered statistically significant.

## 3. Results

### 3.1. Oxidative Status

Results showed that the levels of lipid oxidative product (TBARS and LPO) and protein carbonyls were significantly increased in HD and PD patients compared to the others' stage of CKD (*P* < 0.001) (Table 2). Indeed, TBARS concentrations were 2.4-fold higher in HD, 2-fold higher in PD, and 1.5-fold higher in CKD4 than in CKD1 patients (*P* < 0.001). LPO were also 5-fold higher in HD, 4.2-fold higher in PD, and 4.3-fold higher in CKD4 than in CKD1 patients (*P* < 0.001). Carbonyls were 3.3-fold higher in HD, 3.4-fold higher in PD and 2.4-fold higher in CKD4 than in CKD1 patients (*P* < 0.001). Our results showed a considerable increase on protein carbonyls in PD patients. Indeed, it is important to note that the levels of the latter products were already increased at stage 3 and stage 4 of CKD.

### 3.2. Antioxidative Status

Our results on the antioxidants status were reported in Table 3. Our results showed that SOD activity was decreased by −60% in HD, by −64% in PD, and by −66% in CKD4 than in CKD1 patients (*P* < 0.001). CAT activity was also significantly decreased by −76% in HD, −82% in PD, and −81% in CKD4 than in CKD1 patients (*P* < 0.001). A decrease in GSH-Px activity was noted in HD by −52%, in PD by −64%, and in CKD4 by −65% compared to CKD1 (*P* < 0.001). Similar result was observed for GSH-GR activity, which diminished by −57% in HD, by −66% in PD, and by −62% in CKD4 compared to CKD1 patients (*P* < 0.001).

Vit E concentrations were lower in all groups, especially in HD patients, which lowered by −36% compared to CKD1 (*P* < 0.001). Similar results were obtained for iron and bilirubin concentrations in which both of them decreased in all study groups especially in HD patients. Iron concentration was diminished by −73% in HD patients compared to CKD1 (*P* < 0.001), whereas bilirubin concentration was diminished around −65% in HD and PD patients compared to CKD1 patients (*P* < 0.001). Indeed, patients with severe and advanced stage of CKD had marked decreases in the concentrations of the antioxidants enzymes. Patients also had decreased Vit E, iron, and bilirubin. Further, this decrease was more pronounced in HD and PD patients.

## 4. Discussion

A comparative study was conducted in CKD patients in order to assess lipid peroxidation, protein oxidation, and antioxidant defence during the progression of CKD and to evaluate the effect of dialysis treatment on redox status.

The case-control studies agree that CKD patients have increased OS produced by an imbalance between pro- and antioxidant capacities [18, 19]. This OS is responsible for the peroxidation of macromolecules such as lipids and proteins causing significant damage. Several pathophysiologic explanations have been claimed; some attribute it to malnutrition and hypoalbuminemia having in these cases low availability of “thiol”; others to “uremic status” itself with solute retention that may favor their pathogenicity; and others to the association of comorbid factors such as advanced age, diabetes, and inflammatory and infectious phenomena [20, 21]. Besides, when these uremic patients receive treatment with an extrarenal depurative technique, as in HD, OS is promoted by several reasons among which the usage of low biocompatible synthetic membranes and the lack of ultrapure dialysis water stand out [22].

OS is highly present in CKD patients, and it may contribute directly to the endothelial activation which favors the precocious atherosclerosis seen in these patients. Further investigation is still required in order to estimate the imbalance between antioxidant and oxidant factors, and in particular it is essential to investigate whether different RRT achieve similar levels of OS [18], especially in patients with high risk to develop CVD. Moreover, the effect of different RRT on oxidative status compared to undialyzed patients with different stage of CKD has not been clearly described.

In the study, we found an increase in markers of lipid peroxidation, in which a production of TBARS and LPO was significantly higher already at severe stage of CKD. Furthermore, we noted a significant increase in the production of protein oxidation. Carbonyls were increased already at severe stage of CKD and its production was intensifying in both HD and PD patients. Our results were in agreement with previous findings which associated the increased levels of lipid peroxidation with the severity of coronary atherosclerosis in patients with CKD [23].

Free radicals are the source of lipid peroxidation derived from oxygen, and the first line of defense against them is SOD. Its function is to catalyze the conversion of superoxide radicals to hydrogen peroxide (H_2_O_2_). Indeed, the decreased SOD activity in our study, especially in HD patients, suggests that accumulation of superoxide anion radical might be responsible for increased lipid peroxidation [24]. Glutathione is a tripeptidic thiol found in the inside of all animal cells and likely is the most important cellular antioxidant. Oxidized glutathione (GSSG) is highly toxic to cells so that the organism tends to reduce GSSG to GSH through glutathione reductase. Thus, determining GSH-GR is considered a reliable estimate of the degree of cellular OS [25]. Moreover, GSH-Px is responsible for the most decomposition of lipid peroxide and protects the cell from the deleterious effects of peroxides [24]. H_2_O_2_ in the presence of sufficient CAT activity will be converted to harmless H_2_O and O_2_. Our patients have decreased levels of the four enzymes; this decrease was more important in HD and PD patients. Hence our result explained the reason of the increase in TBARS and LPO levels.

Vitamin E has an antioxidant effect that protects tissue from the oxidative damage [26]. Our investigation confirms the previous studies which found a significant decreased level of vitamin E in advanced stages of CKD patients especially those on HD [27].

Plasma concentrations of the essential metals as iron (Fe) were significantly lower in HD patients than in the other groups of the study population. We found that patients had a strong association between increased plasma OS and decreased concentration of Fe. Long-term HD patients have significantly lower blood concentrations of Fe, and the possible causes of the loss of these essential metals have been studied [28, 29]. Indeed, accumulating FR accompanied reduced tissue concentrations of Fe and induced oxidative damage to brain tissue [30].

Additionally, bilirubin concentration is the primary defense against OS in extracellular fluid results, generated during normal metabolism or introduced in the body by the consumption of dietary products rich in antioxidants [29]. Knowing that our HD patients had dietary restriction intakes that are a part of a renal care, this unbalanced diet contributes to a significant OS.

Moreover, in the HD population, the interaction between dialysis membranes and blood can trigger the release of oxygen-FR and oxidizing agents, such as superoxide anion, hydrogen peroxide, and myeloperoxidase. In turn, these molecules contribute to the oxidation of lipid products, proteins and nucleic acids. This oxidation has several pathophysiological consequences, including enhanced atherogenicity of Ox-LDL, and accelerated demise of circulating erythrocytes, leading to a shorter life span [31, 32].

Our patients were treated by a conventional PD considered as bioincompatible. Repeated and long-term exposure to conventional glucose-based PD fluids plays a central role in the pathogenesis of the functional and structural changes of peritoneal membrane. Low pH, high glucose concentration, high osmolarity and heat sterilization represent major factors of low biocompatibility [33]. Bioincompatibility of PD solutions seems to play a central role in the increase of ROS production [34].

In conclusion, our study showed that impaired renal function and duration of dialysis treatment are associated with increased OS. Consequently decreased antioxidants defence which leads to promote the malnutrition, inflammation, and atherosclerosis (MIA syndrome). Our data suggest that patients subjected to RRT need new methods aimed to reduce intradialytic OS, such as incorporating antioxidant therapy into the dialysis membrane, hemolipodialysis, using of electrolyte-reduced water for dialysate, or using an ultrapure dialysate system to reduce acute phase inflammation.

## Figures and Tables

**Table 1:** Characteristics of the study populations.

	CKD 1 *n* = 28	CKD 2 *n* = 28	CKD 3 *n* = 28	CKD 4 *n* = 18	HD *n* = 40	PD *n* = 25
Age (years)	37 ± 13	55 ± 11	45 ± 15	46 ± 14	42 ± 11	39 ± 15
Weight (kg)	66 ± 12.74	67 ± 16.12	67 ± 13.96	61 ± 13.87	56.44 ± 11.37	68.22 ± 10.55
BMI (Kg/m^2^)	24.07 ± 7.16	25.35 ± 5.63	24.91 ± 3.54	23.63 ± 4.01	23.15 ± 3.02	25.43 ± 3.28
Sex ratio (M/F)	10/18	11/17	10/18	07/11	22/18	06/06
GFR (mL/min)	86.25 ± 23.87	46 ± 6.95	20.31 ± 5.10	10.75 ± 2.56	—	—
Dialysis duration (months)	—	—	—	—	14–109	05–49
Urea (g/L)	0.34 ± 0.15	0.53 ± 0.20	1.05 ± 0.34	1.67 ± 0.82	1.32 ± 0.36	1.04 ± 0.38
Creatinin (mg/L)	10.30 ± 2.89	18.54 ± 5.26	41.75 ± 11.46	70.03 ± 26.15	102.12 ± 29.37	69.36 ± 36.95
Uric acid (g/L)	52.63 ± 11.28	64.10 ± 13.55	80.85 ± 14.11	86.67 ± 20.01	65.55 ± 13.44	68.10 ± 15.62
Total proteins (g/L)	75.17 ± 5.21	74.29 ± 7.52	71.16 ± 11.15	70.86 ± 10.52	69.92 ± 9.60	67.04 ± 7.36
Cholesterol (g/L)	1.68 ± 0.33	1.89 ± 0.29	1.73 ± 0.32	1.74 ± 0.45	2.17 ± 1.58	1.95 ± 0.56
HDL-cholesterol (g/L)	0.53 ± 0.10	0.48 ± 0.09	0.50 ± 0.10	0.44 ± 0.11	0.36 ± 0.11	0.42 ± 0.07
LDL-cholesterol (g/L)	0.93 ± 0.33	1.13 ± 0.22	0.95 ± 0.93	0.94 ± 0.41	1.14 ± 0.51	1.21 ± 0.45
Triglycerides (g/L)	1.17 ± 0.53	1.39 ± 0.55	1.47 ± 0.74	1.30 ± 0.55	1.64 ± 0.90	1.75 ± 0.89
Erythrocytes, ×10^6^/*μ*L	4.72 ± 0.70	4.43 ± 0.59	4.03 ± 0.46	3.25 ± 0.50	3.07 ± 0.73	3.68 ± 0.86
Hemoglobin (g/dL)	13.01 ± 1.36	12.28 ± 1.31	11.46 ± 1.53	8.71 ± 1.37	9.55 ± 1.86	10.38 ± 2.44

BMI: body mass index (weight kg/height m^2^); GFR: glomerular filtration rate. Data are spoken in mean ± SD.

**Table 2:** Oxidative status in the study groups.

	CKD 1	CKD 2	CKD 3	CKD 4	HD	PD	*P*
TBARS (*μ*mol/L)	0.55 ± 0.19	0.62 ± 0.11	0.71 ± 0.08	0.82 ± 0.08	1.36 ± 0.12	1.11 ± 0.24	<0.001
LPO (nmol/mL)	0.34 ± 0.26	0.80 ± 0.39	1.25 ± 0.36	1.46 ± 0.17	1.71 ± 0.43	1.45 ± 0.12	<0.001
Carbonyls (nmol/mg)	0.56 ± 0.15	0.95 ± 0.13	1.04 ± 0.33	1.37 ± 0.36	1.85 ± 0.16	1.92 ± 0.13	<0.001

TBARS: thiobarbituric acid reactive substances; LPO: hydroperoxides. Data are presented as the mean ± SD. Statistically significant differences between all the groups (*P* < 0.001).

**Table 3:** Antioxidative status in the study groups.

	CKD 1	CKD 2	CKD 3	CKD 4	HD	PD	*P*
SOD (U/mL)	81.89 ± 8.38	68.37 ± 2.45	60.23 ± 6.15	54.29 ± 2.94	49.01 ± 3.03	52.53 ± 4.33	<0.001
CAT (U/mL)	62.75 ± 5.42	57.76 ± 7.25	53.52 ± 8.48	51.22 ± 3.66	47.99 ± 5.90	51.92 ± 5.90	<0.001
GSH-Px (U/mL)	7.71 ± 1.01	6.62 ± 0.50	5.23 ± 0.71	5.01 ± 0.82	3.99 ± 1.39	4.94 ± 1.12	<0.001
GSH-GR (U/mL)	1.72 ± 0.06	1.45 ± 0.11	1.34 ± 0.12	1.07 ± 0.19	0.98 ± 0.19	1.13 ± 0.21	<0.001
Vit E (ng/mL)	0.81 ± 0.06	0.62 ± 0.04	0.43 ± 0.05	0.36 ± 0.01	0.29 ± 0.03	0.36 ± 0.01	<0.001
Fe (*μ*g/dL)	81.44 ± 20.45	74.90 ± 8.53	67.00 ± 10.98	61.42 ± 22.08	59.22 ± 16.39	60.90 ± 19.72	<0.001
Bilirubin (mg/L)	6.37 ± 1.80	5.95 ± 1.96	5.50 ± 1.43	5.00 ± 1.41	4.48 ± 1.68	4.40 ± 1.07	<0.001

SOD: superoxide dismutase; CAT: catalase; GSH-Px: glutathione peroxidase; GSH-GR: glutathione reductase; Vit E: vitamin E; Fe: iron. Data are presented as the mean ± SD. Statistically significant differences between all the groups (*P* < 0.001).
